# Population genetic analysis reveals barriers and corridors for gene flow within and among riparian populations of a rare plant

**DOI:** 10.1093/aobpla/plx065

**Published:** 2017-11-22

**Authors:** Tanya H Hevroy, Michael L Moody, Siegfried L Krauss

**Affiliations:** School of Plant Biology, Faculty of Natural and Agricultural Sciences, The University of Western Australia, Crawley, Western Australia, Australia; Department of Biological Sciences, University of Texas at El Paso, TX, USA; Kings Park and Botanic Garden, Botanic Gardens and Parks Authority, Western Australia, Australia

**Keywords:** Dispersal, landscape genetics, metapopulation, riparian systems, Southwest Australian Floristic Region, spatial genetic structure

## Abstract

Landscape features and life-history traits affect gene flow, migration and drift to impact on spatial genetic structure of species. Understanding this is important for managing genetic diversity of threatened species. This study assessed the spatial genetic structure of the rare riparian *Grevillea* sp. Cooljarloo (Proteaceae), which is restricted to a 20 km^2^ region impacted by mining in the northern sandplains of the Southwest Australian Floristic Region, an international biodiversity hotspot. Within creek lines and floodplains, the distribution is largely continuous. Models of dispersal within riparian systems were assessed by spatial genetic analyses including population level partitioning of genetic variation and individual Bayesian clustering. High levels of genetic variation and weak isolation by distance within creek line and floodplain populations suggest large effective population sizes and strong connectivity, with little evidence for unidirectional gene flow as might be expected from hydrochory. Regional clustering of creek line populations and strong divergence among creek line populations suggest substantially lower levels of gene flow among creek lines than within creek lines. There was however a surprising amount of genetic admixture in floodplain populations, which could be explained by irregular flooding and/or movements by highly mobile nectar-feeding bird pollinators. Our results highlight that for conservation of rare riparian species, avoiding an impact to hydrodynamic processes, such as water tables and flooding dynamics, may be just as critical as avoiding direct impacts on the number of plants.

## Teaser

This study assessed the spatial genetic structure of a rare plant (Grevillea) situated along creeks and on floodplains in the Southwest Australian Floristic Region. The landscape features of this unique region and the life-history traits of this species have had profound impacts on its structure. Our results highlight that for conservation of riparian species, avoiding an impact to hydrodynamic processes is critical, as they play a vital role in species gene flow, and consequently, genetic diversity. Understanding this is important for managing threatened species, especially one in an international biodiversity hotspot such as the SAFR in Western Australia.

## Introduction

Gene flow is a key evolutionary force affecting spatial genetic structure within species (Ellstrand 2014). Gene flow in plants occurs through diploid embryos in seeds and haploid gametes in pollen (Holderegger *et al.* 2010). These processes are influenced by the composition of landscapes they inhabit, as well as life-history traits such as pollination, mating systems and vectors for dispersal (Storfer *et al.* 2007; Holderegger *et al.* 2010).

Landscape features impacting gene flow may be striking, for example mountain ranges (Hu *et al.* 2008; Trénel *et al.* 2008), intervening habitats between riparian environments (Kitamoto *et al.* 2005; Mitsui *et al.* 2010) or anthropogenic induced fragmentation (Cegelski *et al.* 2003; Epps *et al.* 2007). However, in many landscapes, barriers to gene flow may be more cryptic, for instance historical vegetation changes (Jacquemyn *et al.* 2006), plant physiology (Nistelberger *et al.* 2015), and/or a combination of both biotic (e.g. pollinator response to habitat features) and abiotic (e.g. wind as a dispersal vector) factors (Holderegger *et al.* 2010). For riparian habitats, landscape features such as rivers, creek lines and floodplains can provide critical corridors that can both facilitate and limit dispersal (Tero *et al.* 2003; Storfer *et al.* 2007; Hu *et al.* 2008). Establishing an understanding of the landscape features that influence spatial genetic structure and gene flow helps to predict and manage potential impacts on rare species from landscape disturbances.

Through spatial analyses of individual genotypes and genetic clusters, landscape features affecting dispersal can be better understood. Clusters can be viewed as genetically divergent groups of individuals where gene flow is inferred to be impeded (Guillot *et al.* 2005; Chen *et al.* 2007; Corander *et al.* 2008). Clustering models applying Bayesian assignment methods have seen a shift from population-based to individual-based analyses (Storfer *et al.* 2010). These methods are especially useful for rare species that are localized with individuals not obviously clustered in discrete populations but rather arranged more continuously, such as in many riparian habitats, and when barriers to gene flow may be more cryptic (Manel *et al.* 2003).

Riparian distributions are typically characterized by largely linear patterns in which gene flow is likely to occur more readily within, rather than among, habitats (Wei *et al.* 2013). For example, seeds dispersed by water (hydrochory) will promote gene flow within but not among riparian corridors. Hydrochory as a force of dispersal can be responsible for influencing spatial genetic structuring of populations along riparian environments at multiple scales (Kudoh *et al.* 2006; Liu *et al.* 2006; Nilsson *et al.* 2010). However, bidirectional pollinator movement, zoochory or wind dispersal can counteract unidirectional downstream dispersal by hydrochory (Tero *et al.* 2005; Markwith and Scanlon 2007). While flooding of riparian environments can facilitate seed dispersal (Kitamoto *et al.* 2005; Nilsson *et al.* 2010), other studies indicate that the impact and consequences for spatial genetic structure are not necessarily predictable (Prentis *et al.* 2004).

**Figure 1.**
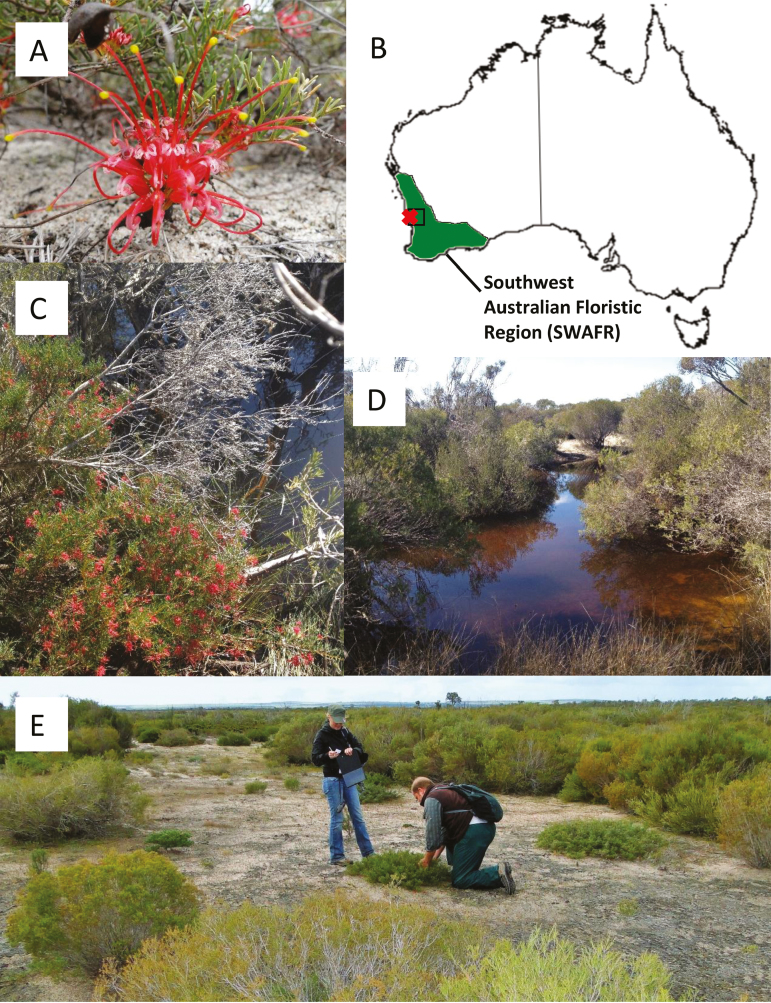
(A) Inflorescence of *Grevillea* sp. Cooljarloo, (B) map illustrating the distribution of *Grevillea* sp. Cooljarloo (X) and the mine site (black square) within the Southwest Australian Floristic Region (SWAFR) in Western Australia, (C and D) *Grevillea* sp. Cooljarloo distribution along creek lines and (E) a floodplain population.

For rare taxa with a narrow endemic range any new limitation to gene flow could be detrimental to the genetic stability and diversity maintained by the species. Therefore, it is important to understand the genetic structure of such species at a landscape scale, where natural features can provide barriers to gene flow even at a small spatial scale. Here, we implement a genetic approach to describe the spatial structure of genetic variation within and among creek line and floodplain populations of a rare plant species, *Grevillea* sp. Cooljarloo (https://florabase.dpaw.wa.gov.au/search/current/37180). This taxon (subsequently referred to as *G.* ‘Cooljarloo’) is listed as Priority 1 (rare and threatened) by the Western Australian Department of Parks and Wildlife (Smith 2013). Although locally abundant, the distribution of *G.* ‘Cooljarloo’ is restricted to creek lines and floodplains (Fig. 1), in a 20 km^2^ area south of Cervantes, in the Southwest Australian Floristic Region (SWAFR), Western Australia (Hopper and Gioia 2004). In particular, we address the influence of landscape features on the genetic patterns found, and how the expansion of a mineral sand-mining operation may impact on conservation plans as a result of further habitat clearing and impact on drainage lines.

## Methods

### Species biology

*Grevillea* ‘Cooljarloo’ is a spreading lignotuborous shrub to 1.5 m on winter-wet sandy soils. Plants typically produce numerous bright red flowers (Fig. 1), which are self-compatible but, like most *Grevillea* (England *et al.* 2003; Roberts *et al.* 2007), are likely to be predominantly outcrossed. The main pollinators are nectar-feeding birds, specifically honeyeaters (Meliphagidae), but marsupials such as the honey possum (*Tarsipes rostratus*) and bees (including the introduced honeybee *Apis mellifera*) are likely to play a role in pollination (Phillips *et al.* 2010). As for most *Grevillea*, *G.* ‘Cooljarloo’ has no obvious adaptations for seed dispersal beyond gravity (Makinson 2000; England *et al.* 2003).

### Sampling strategy and study sites

**Figure 2.**
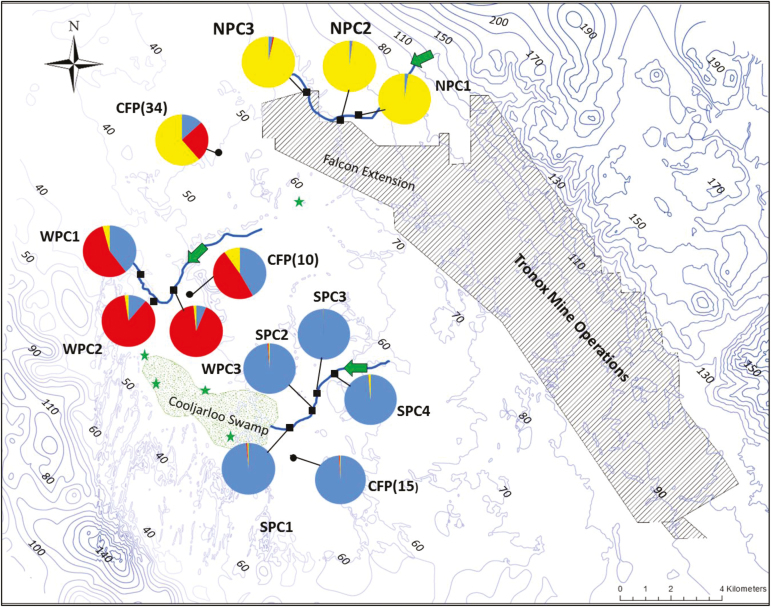
Map of the locations of sampled populations of *Grevillea* sp. Cooljarloo from creeks (black squares) and floodplains (black circles). Sample site names correspond to those in Table 1. Creek lines are drawn onto the map and arrows indicate direction of flow. Numbered contour lines represent elevation, ranging from low (light blue) to high (dark blue) elevation (m a.s.l.). The green stippled area illustrates Cooljarloo Swamp. Stars indicate recorded locations which could not be sampled due to fire and mining operations. Populations are abbreviated for floodplain (CFP) and North (NPC), South (SPC) and West (WPC) creek line. Pie charts indicate the proportion of assignments of sampled populations to each of three genetic clusters, calculated as the mean proportion of membership values for individuals within that population from the Bayesian clustering TESS analysis. Colours correspond to bar plot in Fig. 5C.

A 30-m digital elevation model was constructed from 974 spot heights provided by Tronox Inc. (CAD resources) using ArcGIS 10.1 (ESRI, Redlands, CA, USA) and visualized as contour lines to illustrate elevation changes within the study region (Fig. 2). Creek lines were drawn onto the map based on satellite images and previous landscape mapping provided by Tronox Inc. Leaf samples were collected from three regions within the study area based on GPS points provided by the mine site environmental surveyors, who had previously recorded all populations in the area. Three large creeks occur within the region, here referred to as North (NPC), South (SPC) and West (WPC). Populations within these creeks and three adjoining floodplain populations (CFP) were sampled for this study (Fig. 2).

The three sampled creek lines are found within a *Banksia*-dominated woodland. The west creek line opens up into a floodplain before continuing west into Nambung National Park (Fig. 2). The north creek line is impacted at both ends by agriculture or mining. The southern creek is impacted by agriculture and mining in the east, and enters Cooljarloo Swamp in the south-west. Two sites where *G.* ‘Cooljarloo’ individuals have been previously recorded could not be sampled (Fig. 2; indicated by stars). The first site was recently burnt and contained no live plants. The second site in the north was inaccessible due to current expansions of the Tronox Mine.

Within the creek lines, samples were collected based on a constant sampling fraction (Lowe *et al.* 2009) by defining a 20 m × 20 m perimeter on both sides of the creek every 0.5–1 km. All individuals within the perimeter were sampled, and these were called populations (Table 1). For floodplain populations, every individual from a randomly selected 20 m x 20 m perimeter within each of the three floodplains adjacent to north, south and west creek lines were sampled and these were called populations (Table 1). Each individual sampled was recorded and mapped using GPS (Garmin) and plotted in ArcGIS 10.1 (ESRI, Redlands, CA, USA).

**Table 1.** Sampling information and genetic diversity statistics averaged over nine microsatellite loci for 13 natural populations of *Grevillea* sp. Cooljarloo, including landscape and position of population along creeks (U = upstream and D = downstream), average elevation (m a.s.l.) and coordinates in latitude/longitude. *N* number of individuals genotyped, *N*_A_ total number of alleles detected, *A*_R_ and *pA*_R_ allelic and private allele richness based on the minimum population size of 15 diploid individuals, respectively. *H*_O_ observed heterozygosity, *H*_E_ expected heterozygosity, *F*_IS_ inbreeding coefficient. *Indicates significant deviation Hardy–Weinberg equilibrium based on 10000 permutations and Bonferroni corrected *P* value (*P* < 0.05).

Site	Collection ID	Landscape	Elevation (m)	*N*	*N*_A_	*A*_R_	*pA*_R_	*H*_O_	*H*_E_	*F*_IS_	Coordinates (UTM)	
North	NPC1	Creek line (U)	80	22	5.3	5.1	0.12	0.608	0.691*	0.125	30°38′13.2″S	115°17′20.4″E
	NPC2	Creek line (U)	70	17	5.2	5.0	0.27	0.606	0.684*	0.114	30°38′49.2″S	115°19′30.1″E
	NPC3	Creek line (D)	50	23	5.7	5.0	0.14	0.629	0.677	0.067	30°33′19.8″S	115°18′55.0″E
South	SPC1	Creek line (D)	50	23	5.7	4.9	0.01	0.619	0.663	0.116	30°40′22.4″S	115°18′28.8″E
	SPC2	Creek line (D)	55	15	5.1	4.8	0	0.670	0.727	0.065	30°40′5.52″S	115°18′50.4″E
	SPC3	Creek line (U)	60	28	5.2	3.9	0	0.586	0.644	0.005	30°39′56.1″S	115°18′59.8″E
	SPC4	Creek line (U)	60	15	4.0	4.3	0	0.633	0.628	0.079	30°39′29.5″S	115°19′10.5″E
West	WPC1	Creek line (D)	40	22	6.6	6.0	0.28	0.777	0.739	−0.053	30°37′22.8″S	115°15′32.4″E
	WPC2	Creek line (D)	50	26	6.8	5.9	0.09	0.566	0.723*	0.221	30°37′34.6″S	115°15′0″E
	WPC3	Creek line (U)	60	29	6.7	5.6	0.05	0.696	0.729	0.043	30°37′9.11″S	115°14′42.7″E
	**Mean**		**57.5**	**22**	**5.63**	**5.05**	**0.01**	**0.64**	**0.69**	**0.078**		
	CFP(34)	Floodplain	45	27	6.7	5.6	0.2	0.622	0.695*	0.108	30°34′37.9″S	115°16′41.8″E
	CFP(15)	Floodplain	50	30	6.1	5.2	0.25	0.672	0.696	0.037	30°40′51.3″S	115°18′33.8″E
	CFP(10)	Floodplain	55	28	6.7	5.9	0.15	0.667	0.760	0.118	30°37′33.4″S	115°15′49.7″E
	**Mean**		**50**	**28.33**	**6.50**	**5.57**	**0.20**	**0.65**	**0.72**	**0.090**		

### Genotyping

Genomic DNA was extracted using the modified CTAB extraction method (Doyle 1990). Quantity and quality of DNA were assessed by visualization on a 1.5 % agarose gel and a Nanodrop (Thermo Fisher, Wilmington, DE, USA). Genetic variation was assessed at nine microsatellite loci, which were amplified and scored as reported in Hevroy *et al.* (2013).

Standard genetic diversity parameters, total number of alleles (*N*_A_), observed and expected heterozygosity (*H*_O_ and *H*_E_) and inbreeding coefficient (*F*_IS_), were estimated for each population using GenAlEx 6.5 (Peakall and Smouse 2012). Allelic richness (*A*_R_) and private allelic richness (*p*_R_) were estimated for each population at every locus with HP-rare (Kalinowski 2005) which uses rarefaction to account for differences in sample size. Significance of differences for genetic diversity parameters between creek line and floodplain populations was assessed by *t*-tests. Deviations from Hardy–Weinberg equilibrium and linkage disequilibrium were tested using an adjusted sequential Bonferroni correction (Rice 1989) and 10000 randomizations at the nominal significance level (5 %) in GenAlEx 6.5. MicroChecker 2.2.3 (Van Oosterhout *et al.* 2004) was used to detect presence of null alleles and potential scoring errors.

### Population-based structure analyses

Pairwise population genetic differentiation was estimated by *F*_ST_ with ARLEQUIN V.3.5 (Excoffier and Lischer 2010), and Jost’s *D*_EST_ with SMOGD (Crawford 2010). Genetic distances among populations and spatial collections were also estimated using Nei’s genetic distance (Nei 1972). Ordinations were conducted with all three distance matrices using principal coordinates analysis (PCoA). The total genetic diversity was partitioned into within and among population components, as well as upstream and downstream (based on elevation data), by hierarchical analysis of molecular variance (AMOVA) using ARLEQUIN V.3.5, with 10000 permutations and significance levels set with nominal values of 0.05 following Bonferroni correction. Isolation by distance among populations and among individuals within all three creek lines was evaluated using Mantel tests between log transformed geographic distance and genetic distance [standardized *F*_ST_/(1 − *F*_ST_)] (Rousset 2000) in GenAlEx 6.5. Significance of the correlation was tested using *P*-values from 10000 permutations.

### Individual-based genetic structure analyses

Spatial genetic structure was further analysed using STRUCTURE 2.3.3 (Pritchard *et al.* 2000), which uses a Bayesian algorithm to estimate probability of membership of an individual (*q*) to an assumed genetic cluster, using a Markov Chain Monte Carlo algorithm. Given the restricted range of populations, an admixture ancestry model with correlated allele frequencies was used. The number of potential genetic clusters (*K*) in the data set was tested from 1 to 10, replicated 10 times for 1 million generations and a burn-in of 100000. Optimum *K* was identified using the ∆*K* function of Evanno *et al.* (2005), which is based on second rate change of likelihood function with respect to *K*, and was calculated using STRUCTURE HARVESTER (Earl and von Holdt 2012).

Given the geographic clustering of populations, we also employed a Bayesian assignment method that explicitly incorporated geographic prior distributions (Storfer *et al.* 2007). TESS 2.3 (Chen *et al.* 2007) implements a Bayesian clustering algorithm that uses a hidden Markov random fields model and allows for a detailed admixture analysis. This method is based on the spatial autocorrelation assumptions that allele frequencies at a geographic site are more likely to be similar to allele frequencies at neighbouring sites than at distant sites (François and Durand 2010). This method provides better estimates of ancestry coefficients when levels of ancestral population divergence are low (Durand 2007), which is a potential issue with our data.

The degree of spatial dependence incorporated in the analyses is determined by a parameter (ψ), which represents the strength of spatial autocorrelation, ranging from 0 to 1. In this analysis, the spatial parameter was set ψ = 0.6, as recommended by the authors (Chen *et al.* 2007). Each run was performed under the admixture model using 50000 cycles with a burn-in of 10000 for *K* = 2–8 with 100 iterations for each *K*. Based on the 10 highest deviance information criterion (DIC) values for each *K*, the most likely number of clusters (*K*) was determined from where the DIC values plateau (Chen *et al.* 2007). The proportion of membership of each individual to given clusters at the optimal *K* (*q*_*i*_) was obtained to illustrate these values. Similarity among different runs was assessed using the similarity coefficient (*H*) in CLUMPP 1.1.2 (Jakobsson and Rosenberg 2007) for both replicates of STRUCTURE and TESS runs at optimal *K*.

## Results

### Landscape elevation

Elevation within the study site ranged from 30 to 205 m. Areas where *G.* ‘Cooljarloo’ populations were mapped and sampled had an average elevation of 51 ± 0.5 m (37–79 m), which was significantly lower than the average elevation of 91 ± 2.3 (52–91 m) (*P* < 0.001) in habitats where *G.* ‘Cooljarloo’ was not found. Elevational differences affected the habitat types (creek lines, floodplains, sandplains), which reflect the presence or absence of *G.* ‘Cooljarloo’.

### Genetic diversity

The nine polymorphic loci amplified a total of 102 alleles across 355 individuals. Only locus GM13 exhibited potential null alleles. Genetic analyses without this marker did not generate significant differences in results compared to the full data set, so it was included in analyses to increase robustness. Mean estimates (and range) of genetic parameters across nine loci were: number of alleles 5.2 (range = 3.6–6.2), allelic richness 5.1 (3.9–5.9), observed heterozygosity 0.64 (0.58–0.77) and expected heterozygosity 0.69 (0.63–0.76). *F*_IS_ deviated significantly from zero within five populations (Table 1), with fewer heterozygotes than expected. Private alleles were largely absent in the SPC creek line populations, and relatively higher in the floodplain and NPC creek line populations (*P* < 0.001). Mean private allelic richness was significantly greater (*t* = 2.3; *P* < 0.05) for floodplain populations (*p*_R_ = 0.2) than creek line populations (*p*_R_ = 0.1) (Table 1). All other genetic diversity parameters were not significantly different.

### Population-based genetic structure

Mean (and range) pairwise *F*_ST_ across all populations was 0.087 (0.028–0.178), mean pairwise *D*_EST_ was 0.215 (0.072–0.422) (Table 2), and both parameters showed similar patterns of divergence. There was significant genetic differentiation for all pairwise population comparisons except NPC1/NPC2, and SPC1/SPC4 (Table 2). Genetic differentiation was higher for inter-creek population pairs than for intra-creek population pairs (mean *F*_ST_ = 0.112 (inter), 0.064 (intra), *P* = 0.024; mean *D*_EST_ = 0.273 (inter), 0.137 (intra), *P* = 0.014). The highest pairwise *F*_ST_ value was 0.178 (NPC2/SPC3) and the lowest was 0.027 (WPC2/WPC3), which reflected geographic distance (12.2 and 0.9 km, respectively). The highest *D*_EST_ value was 0.422 (NPC1/SPC2) and the lowest was 0.072 (NPC1/NPC2) again reflecting geographic distance (11.7 and 0.3 km, respectively). Pairwise *F*_ST_ for intra-creek population pairs for each individual creek was 0.065 (NPC), 0.060 (SPC) and 0.054 (WPC), and *D*_EST_ was 0.145 (NPC), 0.097 (SPC) and 0.170 (WPC). In addition, pairwise *F*_ST_ for inter-creek comparisons when we compare one creek to another was 0.105 (NPC vs. SPC), 0.088 (NPC vs. WPC) and 0.138 (SPC vs. NPC), and *D*_EST_ comparisons are 0.233 (NPC vs. SPC), 0.245 (NPC vs. WPC) and 0.339 (SPC vs. NPC).

**Table 2.** Pairwise *D*_EST_ and *F*_ST_ between 10 creek line and 3 floodplain populations of *Grevillea* sp. Cooljarloo. Upper diagonal: Jost (2008) *D*_EST_ and lower diagonal: mean *F*_ST_ values. Values are significantly different from zero for all population comparisons at the 5 % level after Bonferroni correction for multiple comparisons except those indicated in bold.

	CFP10	CFP15	CFP34	NPC1	NPC2	NPC3	SPC1	SPC2	SPC3	SPC4	WPC1	WPC2	WPC3
CFP10		0.179	0.094	0.191	0.199	0.185	0.195	0.168	0.230	0.214	0.182	0.078	0.164
CFP15	0.054		0.152	0.292	0.325	0.235	0.097	0.235	0.175	0.211	0.156	0.229	0.201
CFP34	0.030	0.041		0.126	0.156	0.201	0.145	0.193	0.235	0.186	0.250	0.101	0.159
NPC1	0.057	0.067	0.061		**0.072**	0.177	0.268	0.422	0.357	0.288	0.392	0.165	0.271
NPC2	0.073	0.119	0.080	**0.032**		0.186	0.270	0.384	0.395	0.366	0.359	0.150	0.218
NPC3	0.086	0.098	0.087	0.070	0.093		0.272	0.398	0.320	0.331	0.228	0.184	0.239
SPC1	0.061	0.041	0.051	0.071	0.123	0.113		0.095	0.092	0.091	0.179	0.199	0.187
SPC2	0.057	0.110	0.084	0.146	0.166	0.175	0.046		0.074	0.144	0.200	0.179	0.179
SPC3	0.089	0.093	0.109	0.116	0.178	0.148	0.047	0.060		0.084	0.227	0.297	0.263
SPC4	0.087	0.095	0.094	0.100	0.176	0.150	0.045	0.103	0.058		0.308	0.299	0.278
WPC1	0.049	0.063	0.065	0.090	0.112	0.091	0.068	0.073	0.096	0.120		0.227	0.204
WPC2	0.037	0.082	0.055	0.071	0.074	0.097	0.076	0.076	0.138	0.146	0.071		0.078
WPC3	0.057	0.074	0.060	0.077	0.082	0.095	0.081	0.101	0.136	0.147	0.064	**0.027**	

Analysis of molecular variance partitioned a small, but significant, amount of the total genetic variation among the three geographic clusters (4.5 %), among populations within geographic clusters (4.6 %) and among individuals within populations (4.1 %), with the remainder (86.8 %) partitioned within individuals (Table 3). When applied to the creek lines alone, 89.4 % of total genetic variation was partitioned within populations, 5.5 % partitioned among the three creek lines and 5.1 % among populations within creek lines (data not shown). When AMOVA was applied to downstream and upstream populations, no significant amount of variation was partitioned among them (2.0 %, *P* = 0.02; Table 3).

**Table 3.** AMOVA for *Grevillea* sp. Cooljarloo populations at various hierarchical levels. Clusters correspond to the unique genetic clustering from TESS spatial analyses. Clusters include both creek line and corresponding floodplain populations (NPC + CFP34, WPC + CFP(10) and SPC + CFP(10)). Refer to Table 1 for list of upstream and downstream populations.

Source of variation	*d*.*f*.	Sum of squares	Percentage of variation	*P*
Among clusters	2	84.8	4.1	<0.001
Among populations within clusters	13	124.8	4.5	<0.001
Among individuals within populations	349	1072.4	4.6	<0.001
Within individuals	365	1014.5	86.8	<0.001
Upstream vs. downstream	1	33.4	2.0	0.02
Within streams	256	2072.5	98	<0.001

**Figure 3.**
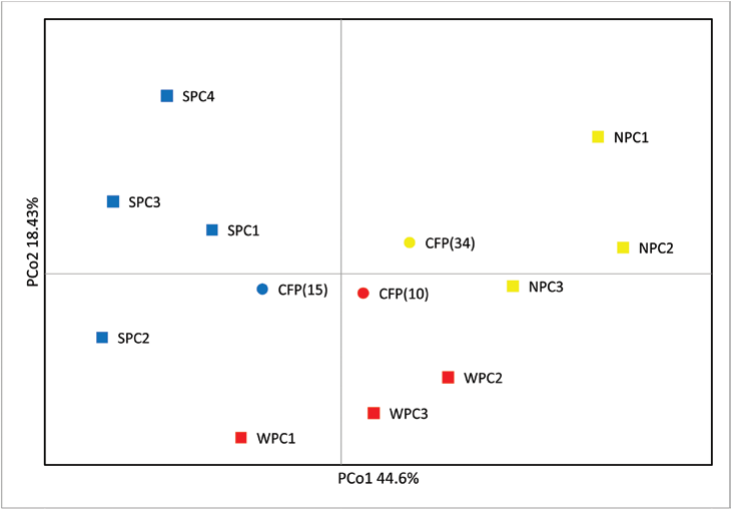
A principal coordinates (PCoA) plot of the first two components calculated using Nei’s *D* genetic distance measure between creek line populations and floodplain populations of *Grevillea* sp. Cooljarloo. Squares and circles refer to creek line populations and floodplain populations, respectively. Colours correspond to TESS-inferred optimal *K* clusters, and labels correspond to populations in Table 1. Note the positions reflecting genetic similarity of the three floodplain populations (CFP).

**Figure 4.**
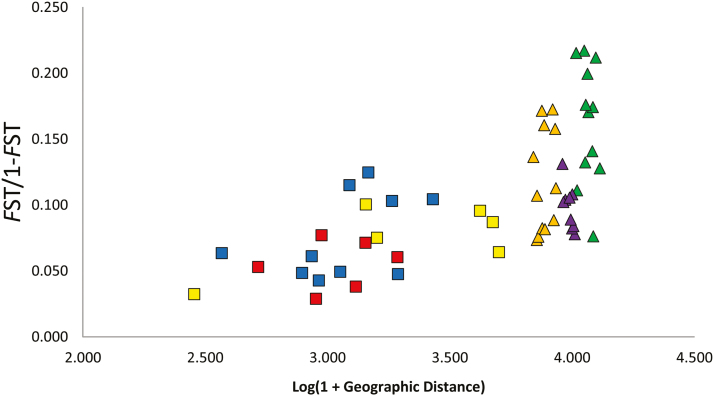
Scatter plot of pairwise population genetic differentiation (*F*_ST_/(1 − *F*_ST_)) against log geographic distance among populations of *Grevillea* sp. Cooljarloo. Comparisons between populations within creek lines are illustrated by coloured squares corresponding to TESS-inferred optimal *K* clusters. Comparisons between populations among creek lines are illustrated by triangles in green (NPC vs. SPC), purple (NPC vs. WPC) and orange (SPC vs. WPC).

Near identical ordinations were generated from each of the three distance matrices. The first two principal axes in the PCoA of Nei’s *D* distance (Fig. 3) explained 44.6 and 18.43 % of the total genetic variation, respectively. The PCoA showed the NPC, SPC and WPC populations clustered separately, and the floodplain populations clustered together in between creeks. The PCoAs of *F*_ST_ and *D*_EST_ genetic distance explained 34.8 and 18.8 %, and 34.2 and 19.2 %, respectively. The association of pairwise population differentiation (*F*_ST_/(1 − *F*_ST_)) against logarithmic geographic distance was significant when calculated across all sampled populations (*r*^2^ = 0.230, *P* = 0.0001; Fig. 4), indicating overall isolation by distance. However, the association between genetic distance and geographic distance among individuals within each creek line (NPC, SPC or WPC) was not significant (*r*^2^ = 0.008; *r*^2^ = 0.001; *r*^2^ = 0.033, *P* > 0.05 in each case).

### Individual-based genetic structure

**Figure 5.**
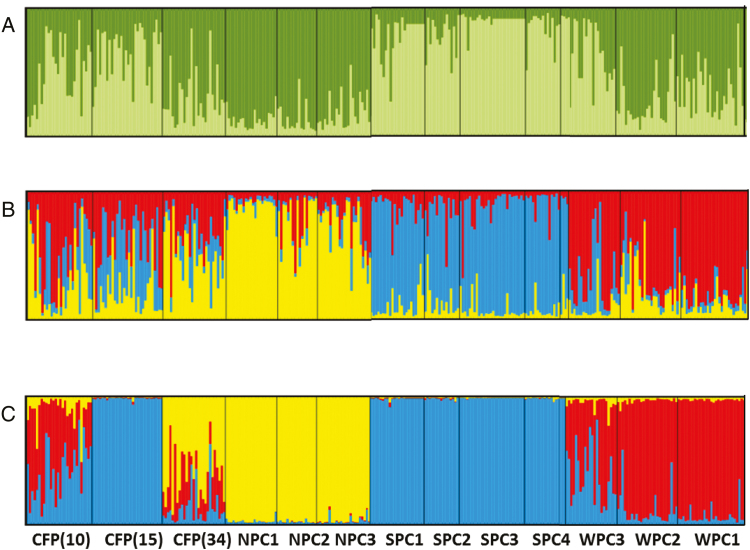
Bar plots representing admixture coefficients for *Grevillea* sp. Cooljarloo populations from assignment tests performed (A) in STRUCTURE with *K* = 2; and (B) in STRUCTURE *K* = 3; and (C) with spatial data in TESS with *K* = 3. Each vertical bar represents an individual, and bars are divided into proportions based upon the probability of assignment to each population.

There was no spatial genetic structure identified within creek lines from the individual-based Bayesian analyses but there was evidence for significant genetic structure between the creek lines. The plot of ∆*K* against *K***[see**Supporting Information—Fig. S1**]** values from STRUCTURE indicated two genetic clusters were optimal (Fig. 5A and B), comprising northern and southern populations, respectively.

The distribution of genetic clusters across the creek lines and floodplains as assigned by TESS is illustrated in Fig. 2, and the individual membership proportions are illustrated in Fig. 5C. The DIC values indicated the optimal number of clusters as three **[see**Supporting Information—Fig. S1**]**. When using admixture analyses, the DIC values for three clusters dropped considerably (no-admixture lowest run = 26979, admixture lowest run = 20115) suggesting that the admixture model had a better fit (Row *et al.* 2010). Chen *et al.* (2007) suggested evaluating posterior probabilities when using spatial components in assignment testing, and to base success on ability of the programs to assign number of individuals to a cluster with membership above 0.8. Under this criterion, TESS assigned 80 % of individuals to a cluster at *K* = 3, outperforming STRUCTURE, which assigned 57 % at *K* = 2 and 47 % at *K* = 3. Individuals from SPC populations had the greatest mean proportion of membership (*q*_*i*_ = 0.98), followed by NPC (*q*_*i*_ = 0.97) and WPC (*q*_*i*_ = 0.78). Two CFP populations, CFP(10) and CFP(34), showed high levels of admixture. CFP (10) showed highest identity with SPC (*q*_*i*_ = 0.42) and WPC populations (*q*_*i*_ = 0.48), whereas CFP (34) had highest identity with NPC (*q*_*i*_ = 0.61) and SPC (*q*_*i*_ =0.26) populations. CFP (15) was clustered with SCP populations (*q*_*i*_ = 0.99).

## Discussion

*Grevillea* ‘Cooljarloo’ is restricted to creek lines and floodplains with an average elevation of 51 m a.s.l., and is absent from intervening habitats that average 91 m in elevation. These landscape features strongly influence the distribution and spatial structure of genetic variation for this species. Spatial genetic analyses supported a conclusion of three genetic clusters that largely corresponded to the disjunct regional distribution of *G.* ‘Cooljarloo’ in northern, southern and western creek lines and floodplains. A significant isolation by distance relationship was associated with comparisons among creek lines, but not within creek lines. Similar levels of genetic differentiation across similarly small spatial scales have been found in many other riparian plant species (Gornall *et al.* 1998; Prentis *et al.* 2004; Jacquemyn *et al.* 2006; Prentis and Mather 2008) suggesting that extensive dispersal is not generally a feature of these systems (as might be expected from hydrochory).

The relatively even distribution of alleles, allelic richness, moderately high heterozygosity and weak genetic differentiation within creek lines does not support a largely unidirectional, source–sink relationship between upstream (source) and downstream (sink) populations (again, as might be expected from hydrochory). This conclusion is consistent with some previous tests of unidirectional dispersal in other riparian systems, where the absence of downstream accumulation of genetic diversity is proposed to be due to upstream seed dispersal through zoochory and higher seed recruitment opportunities in upstream habitats due to density dependence of recruitment (Honnay *et al.* 2010; Nillson *et al.* 2010). *Grevillea* ‘Cooljarloo’ has no obvious mechanism for seed dispersal by zoochory, and hydrochory appears to play a minor role in dispersal. Rather, we suggest our results reflect limited, predominantly gravity, dispersal of seeds and wide bi-directional movement of pollen along creek line populations by highly mobile nectar-feeding bird pollinators (discussed further below).

The mean genetic differentiation among populations within creeks (*F*_ST_ = 0.060) was significantly lower than that for pairs of populations among creeks (*F*_ST_ = 0.110), which indicates that landscape features among creek lines inhibit gene flow relative to within creek lines. However, the very wide range of pairwise population genetic distance values between creek lines (*F*_ST_ = 0.032–0.178), despite an overall similarity in geographic distance within creek line populations (Fig. 4), indicates other factors in addition to distance are influencing gene flow here. In particular, floodplain populations showed surprisingly high genetic similarity (*F*_ST_ = 0.030–0.054), much higher than those among creek lines (mean *F*_ST_ = 0.110), despite similar geographic distances. These results suggest that floodplains may be a landscape feature facilitating gene flow independently of distance, and that the non-floodplain landscape between creek lines acts as barriers to gene flow. These results suggest that floodplain flooding events, although less frequent than creek line flow events, provide for more effective and extensive dispersal of seeds. It may also suggest that pollinators are more mobile across floodplains than across creek lines (discussed further below).

Unlike the creek line populations, the admixture evident in the west and north (but not the south) floodplain populations suggests a directional pattern of dispersal to these habitats. This pattern suggests a source–sink relationship from multiple creek line populations into these floodplain populations. Given that multiple sources are involved, the classic source–sink model predicting lower genetic diversity in sink populations coming from a single succession of source populations (Tero *et al.* 2003) is not evident. Instead, these floodplain populations on average possess more private alleles and similar levels of heterozygosity and allelic richness to creek line populations. Gene flow to floodplains is likely induced by occasional flooding of creek lines and maintained by subsequent animal pollination. South-west Australia experiences a Mediterranean type climate, where the bulk of rainfall occurs through winter and spring prior to seed release by *G.* ‘Cooljarloo’. Summers typically experience very low rainfall, but high intensity and short duration storms, derived from tropical airflows, which can lead to episodic recruitment events as floodwaters disperse seed, provide moist seedbeds and create gaps in the vegetation (Pettit *et al.* 2001). Flooding has been shown to play an important role in seed dispersal in many riparian studies (Gornall *et al.* 1998; Kitamoto *et al.* 2005; Kudoh *et al.* 2006; Van Looy *et al.* 2009).

Pollinators, and in particular highly mobile nectar-feeding honeyeater birds, are likely to play a critical role in extensive multidirectional gene flow by pollen in *G.* ‘Cooljarloo’. Bird assemblages in riparian habitats in Australia tend to be significantly greater in richness, diversity and abundance than adjacent non-riparian sites (Palmer and Bennett 2006). This includes honeyeaters, which are major pollinators for many *Grevillea* species (Ford *et al.* 1979; Celebrezze and Paton 2004). Bird pollination can result in far more extensive pollen movement than from insect pollination (Krauss *et al.* 2009; Phillips *et al.* 2010; Krauss *et al.* 2017). We suggest that wide pollen dispersal may act to erode spatial genetic structure arising from predominantly gravity dispersal of seed within creek lines, but this requires further testing through, for example, paternity assignment studies (Krauss *et al.* 2009).

## Conclusions

The distribution of *G.* ‘Cooljarloo’ is associated with lower elevation habitats that are characterized by floodplains and creek lines. These landscape features strongly influence the structure of genetic variation within and among populations within and among creek lines, where distance is a driver of weak genetic structure within creek lines, but landscape features drive genetic differentiation among creek lines. However, our results also demonstrated a complexity that is consistent with a metapopulation model of dispersal in riparian systems (Tero *et al.* 2003; Prentis and Mather 2008; Honnay *et al.* 2010), where geographic distance alone is a poor predictor of genetic distance between populations, and where some, but not all, floodplain populations displayed a surprisingly high level of admixture. The floodplain result suggests a critical role of irregular flooding events in the spatial distribution of genetic variation in this riparian species. Connectivity relating to flood events is an important issue with regards to climate change, as the average rainfall in the south-west of Western Australia has been decreasing over the last 50 years (Bates *et al.* 2008). This suggests the possibility of a contracting distribution for the riparian *G.* ‘Cooljarloo’, due not only to landscapes altered by a changing climate, but also because of a reduced capacity for seed dispersal. These water relationships, including flooding, may also be a critical determinant involved in overcoming seed germination inhibitors (Ma *et al.* 2015) and enabling germination and seedling survival, critical issues requiring further research.

Genetic diversity underpins evolutionary potential of a species and is a measure of population fitness (Spielman *et al.* 2004). Given the strength of genetic differentiation among the few existing *G.* ‘Cooljarloo’ populations across creek lines, the loss of any population will have a significant impact on total genetic diversity. NPC populations in particular are under threat of impact from mining (Fig. 2). These populations have relatively high levels of private alleles and show significant differentiation from other creek line populations, indicating a disproportionally high loss of species genetic diversity with the loss of these populations. While overall plant numbers are plentiful in the region, maintaining the genetic structure and metapopulation processes identified through this research will have an important role in preserving the long-term potential of this taxon. Here, avoiding an impact to hydrodynamic processes, such as water tables and flooding dynamics, is just as critical as avoiding direct impacts on the number of plants.

## Supporting Information

The following additional information is available in the online version of this article—

**Figure S1.** Likely number of genetic clusters in *Grevillea telemannaniana* ssp. Cooljarloo data set from spatial analyses programs based on (A) delta *K* in STRUCTURE, *K* = 2 and (B) deviance information criterion (DIC) in TESS, *K* = 3.

Supporting Information Figure S1

## Sources of Funding

T.H.H. was supported by an Australian Postgraduate Award (APA) and the University Postgraduate Award (UPA) from the University of Western Australia. This work was supported in part by a contract to M.L.M. from Tronox Limited.

## Contributions by the Authors

All authors have made a substantial contribution to the paper.

## Conflict of Interest

None declared.
